# Viral shedding in children infected by pandemic A/H1N1/2009 influenza virus

**DOI:** 10.1186/1743-422X-8-349

**Published:** 2011-07-13

**Authors:** Susanna Esposito, Cristina Daleno, Fausto Baldanti, Alessia Scala, Giulia Campanini, Francesca Taroni, Emilio Fossali, Claudio Pelucchi, Nicola Principi

**Affiliations:** 1 Department of Maternal and Pediatric Sciences, Università degli Studi di Milano, Fondazione IRCCS Ca' Granda Ospedale Maggiore Policlinico, Milan, Italy; 2 Molecular Virology Unit, Fondazione IRCCS Policlinico San Matteo, Pavia, Italy; 3 Department of Epidemiology, Istituto di Ricerche Farmacologiche Mario Negri, Milan, Italy

## Abstract

**Background:**

The aim of this study was to investigate viral shedding in otherwise healthy children with pandemic A/H1N1/2009 influenza in order to define how long children with pandemic A/H1N1/2009 influenza shed the virus, and also plan adequate measures to control the spread of the disease within households.

**Findings:**

In 74 otherwise healthy children with pandemic A/H1N1/2009 influenza, nasopharyngeal swabs were taken for virus detection upon hospital admission and every two days until negative. The nasopharyngeal swabs of all of the children were positive for pandemic A/H1N1/2009 influenza virus in the first three days after the onset of infection, and only 21.6% and 13.5% remained positive after respectively 11 and 15 days. No child was positive after more than 15 days. Viral load also decreased over time, and was not associated with patient age or the risk of pneumonia. Those who shed the virus for ≥ 9 days were not at any increased risk of suffering from more severe disease in comparison with those who shed the virus for a shorter time, but their households experienced a significantly higher number of influenza-like illness during the two weeks after the onset of the initial disease (72.3% *vs *41.4%; p < 0.05).

**Conclusions:**

Regardless of their age, healthy children can shed pandemic A/H1N1/2009 influenza virus for up to two weeks after illness onset, and the households of the children who shed the virus for ≥ 9 days suffered a higher number of influenza-like illness in the two weeks following the onset of the first disease. This could suggest that when a completely unknown influenza virus is circulating, isolation period of infected children has to be longer than the 7 days recommended for the infections due to seasonal influenza viruses.

## Findings

The length of time an influenza infection is contagious is mainly related to the amount and duration of virus shedding [1]. In the case of seasonal influenza, it has been shown that shedding generally coincides with symptom onset, peaks on the second day, and generally lasts for about one week [2]. However, this duration is greatly affected by age and may be longer in young children, thus justifying the assumption that pediatric patients are the main cause of the spread of influenza in the community [3].

The pediatric data concerning the shedding of the novel pandemic A/H1N1/2009 influenza virus are not conclusive because some studies seem to indicate that the virus is shed for a longer time in younger patients than in adults [4], and others that the shedding is not age-related [1,5]. The aim of this study was to investigate viral shedding in otherwise healthy children with pandemic A/H1N1/2009 influenza in order to define how long children with pandemic A/H1N1/2009 influenza shed the virus, and plan adequate measures to control the spread of the disease within households.

This study was approved by the Institutional Review Board of the Fondazione IRCCS Ca' Granda Ospedale Maggiore Policlinico, Milan, Italy. The patients were enrolled by the Department of Maternal and Pediatric Sciences of the University of Milan during the peak period of pandemic A/H1N1/2009 influenza (from 1 November to 15 November 2009) after both parents or the legal guardian of the children had given their written informed consent; the older children were asked to give their assent.

The study involved otherwise healthy subjects aged less than 15 years without any underlying chronic severe disease who attended our Emergency Room or were hospitalised because of an influenza-like illness as defined by the Italian Ministry of Health http://www.ministerosalute.it. Only the children whose symptoms had appeared in the 48 hours preceding hospital attendance were enrolled. Patients with serious complications related to pandemic A/H1N1/2009 influenza who required antiviral treatment were also excluded.

After collecting data concerning the patients' previous clinical history and the exact time of the onset of signs and symptoms of A/H1N1/2009 influenza (including fever), all of the children underwent a complete physical examination and were divided into disease groups on the basis of signs and/or symptoms using well-established criteria (i.e., common cold, acute pharyngotonsillitis, acute otitis media, croup, acute bronchitis, acute wheezing, radiographically-confirmed pneumonia) [6]. The children with radiographically-confirmed pneumonia (all of whom required hospitalisation) were considered separately from those who were not hospitalized and had evidence of upper respiratory tract symptoms or lower respiratory tract symptoms but without evidence of pneumonia.

Upon enrolment, a nasopharyngeal sample for the diagnosis of pandemic A/H1N1/2009 influenza infection was collected. A further swab was taken from all of the positive cases on the third day after the onset of disease and afterwards every two days until two were negative. During the collection of the respiratory secretions, the medical history of each child was re-evaluated (in the outpatient clinic if they had been discharged or in the ward if they were hospitalised), and information was obtained about the occurrence of influenza-like illness (ILI) and the related morbidity in their households. At this regard, only subjects permanently living at home together with the infected child were considered and in case of hospitalization of the enrolled child only those who had taken care daily of the patient during the whole period of the stay in the hospital.

All of the nasopharyngeal samples were collected using a pernasal flocked swab, and stored in a tube of UTM-RT (Kit Cat. No. 360c, Copan Italia, Brescia, Italy). Viral RNA was extracted by means of a Nuclisens EasyMAG automated extraction system (bioMeriéux, Bagno a Ripoli, Florence, Italy), as previously described [7]. Real-time polymerase chain reaction (real-time PCR) was used to identify influenza A and B viruses using previously validated methods [7]. In the case of influenza A-positive samples, the WHO/CDC protocol was used to characterise those positive for pandemic A/H1N1/2009 influenza [8].

A plasmid containing the corresponding target viral sequence was used to quantify viral load in the A/H1N1/2009-positive samples. Ten-fold plasmid serial dilutions ranging from 5 to 5 × 10^7 ^input copies were prepared to generate calibration curves, and run in parallel with the tested samples. The cycle threshold values of each dilution were measured in duplicate and plotted against the logarithm of their initial quantities, and the copy numbers in each clinical sample were derived from the regression line. The quantitative results were expressed as RNA copy number/mL of nasopharyngeal swab following data multiplication by 50. In order to evaluate reproducibility, intra- and inter-assay standard deviations (SDs) and coefficients of variation were calculated for each standard concentration within and between the individual PCR runs.

Mean viral shedding was calculated and plotted by year of age, a diagnosis of pneumonia, and the occurrence of acute respiratory illness in at least one family member. Wilcoxon's rank-sum test was used to assess the between-group differences. In the case of categorical data, the groups were compared using contingency table analysis with the χ^2 ^or Fisher's exact test, when appropriate. All of the analyses were two-tailed, and *p *values of 0.05 or less were considered statistically significant.

Of the 74 children enrolled (36 males; mean age ± SD, 5.2 ± 4.9 years), 44 (59.4%) were hospitalised because of pneumonia and classified as suffering from severe disease. Table 1 shows the mean viral loads (log_10 _cp/mL) and the number of children shedding virus at different times after the onset of pandemic A/H1N1/2009 influenza. The nasopharyngeal swabs of all of the children were positive for pandemic A/H1N1/2009 influenza virus in the first three days after the onset of infection, but the number of positive samples decreased over time and only 21.6% and 13.5% remained positive after respectively 11 and 15 days regardless of patient age. No child was positive after more than 15 days. Fever disappeared 3-4 days after enrolment in both the hospitalised and non-hospitalised patients regardless of viral shedding.

**Table 1:** Mean viral load (log_10 _cp/mL) and number of children shedding virus at different times after onset of pandemic A/H1N1/2009 influenza

	Days after onset								
	
	1-2	3	5	7	9	11	13	15	17
Mean viral load (log_10 _cp/mL ± SD)	8.19 ± 1.41	7.30 ± 1.48	7.00 ± 1.23	3.90 ± 2.83	2.12 ± 3.05	1.09 ± 2.32	1.00 ± 1.66	0.87 ± 2.47	0
Children shedding virus, No. (%)	74 (100.0)	74 (100.0)	57 (77.0)	42 (59.1)	35 (47.3)	16 (21.6)	14 (18.9)	10 (13.5)	0 (0.0)

Viral load also decreased over time, and was not associated with patient age. Moreover, no relationship was found between duration of viral shedding and risk of development of pneumonia because the percentage of children who shed the virus for ≥ 9 days and were admitted to the hospital for pneumonia was similar to that of those who shed the virus for a shorter time and were hospitalized for pneumonia (68.5% *vs *51.2%; p > 0.05). However, households of children with prolonged shedding (≥ 9 days) experienced a significantly higher number of ILI during the two weeks after the onset of the initial disease than those of children who shed the virus for less than 9 days (72.3% *vs *41.4%; p < 0.05).

Our findings show the natural viral shedding profile of children infected with pandemic A/H1N1/2009 influenza virus because none of the enrolled children received any antiviral drug capable of interfering with virus replication during the observation period. Moreover, as only otherwise healthy children were enrolled, they add useful information concerning the real period during which most children spread the infection in the community.

Regardless of their age, healthy children can shed pandemic A/H1N1/2009 influenza virus for up to two weeks after illness onset. Furthermore, the shedding can persist for some days after the cessation of fever. As has been observed in other studies [1,2,9], viral load significantly decreased over time, varying from about 8 log_10 _cp/mL in the first days to more than 3 log_10 _cp/mL in the second week. All of our data seem to suggest that the World Health Organisation (WHO) guidelines for the clinical management of human infection with pandemic A/H1N1/2009 influenza virus should be considered cautiously [10]. The WHO guidelines indicate that patients with pandemic influenza symptoms should be precautionarily isolated for seven days after the onset of illness or 24 hours after the resolution of fever which, on the basis of our findings, seems to be too short a period to avoid the risk of spreading the infection. Furthermore, the significantly greater number of ILI diagnosed in the households of children who shed the virus for ≥ 9 days suggests that prolonged shedding increases the risk. This could mean that, in order to reduce the risk of transmission, a period of self-isolation of at least 10 days may be wiser, particularly when an infected child may come into contact with vulnerable subjects such as pregnant women, newborns or immunocompromised people. However, in order to validate our observation on viral shedding further studies carried out in a bigger cohort of children have to be performed.

In our study we evaluated viral shedding by identifying viral RNA in nasopharyngeal secretions on the basis of real-time PCR and because this method may amplify viral RNA from non-viable virus particles [9] the true infection period could have been not precisely evaluated. Only classical virological methods (cell cultures and the determination of the 50% tissue culture infectious dose) can give exact information concerning the amount replicating viruses really shed by the patient.

Unlike what was expected on the basis of data collected in subjects with seasonal influenza, the duration of pandemic A/H1N1/2009 influenza virus shedding was not related to age because there was no difference between the children in the different age groups. Children with seasonal influenza shed viruses for longer than adults because their lower pre-existing immunity fails to limit viral replication [1]. However, epidemiological data show that no pediatric patient should have any pre-existing immunity against a new virus such as the pandemic A/H1N1/2009 influenza virus [11]. This supports our finding of no age-related difference in the shedding period.

In conclusion, our study confirms that, when a completely new influenza virus such as that present in a pandemic begins to circulate, every precaution to limit the spread of the infection should be taken regardless of patient age and isolation period of infected children has to be longer than the 7 days recommended for the infections due to seasonal influenza viruses.

## List of abbreviations

(ILI): Influenza-like illness; (PCR): polymerase chain reaction; (SD): standard deviation; (WHO): World Health Organisation.

## Competing interests

The authors declare that they have no competing interests.

## Authors' contributions

SE and NP designed the study and co-wrote the manuscript. CD, FB, AS and GC carried out the real-time PCR and quantification of viral load. FT collected the swabs. EF visited the patients. CP performed the statistical analysis. All authors read and approved the final manuscript.
